# Current estimates of T cell kinetics in humans

**DOI:** 10.1016/j.coisb.2019.10.002

**Published:** 2019-12

**Authors:** Derek C. Macallan, Robert Busch, Becca Asquith

**Affiliations:** 1 Institute for Infection and Immunity, St George's, University of London, London, UK; 2 Department of Life Sciences, University of Roehampton, London, UK; 3 Department of Infectious Disease, Imperial College London, London, UK

**Keywords:** Lifespan, Half life, Stable isotope labelling, Proliferation rate, Death rate, T cell, Human, *In vivo*, Best estimates, Naive, Stem cell-like memory, Central memory, Effector memory, Dynamics, Kinetics, Mathematical model

## Abstract

Stable isotope labeling is a generally applicable method of quantifying cell dynamics. Its advent has opened up the way for the quantitative study of T cells in humans. However, the literature is confusing as estimates vary by orders of magnitude between studies. In this short review we aim to explain the reasons for the discrepancies in estimates, clarify which estimates have been superseded and why and highlight the current best estimates. We focus on stable isotope labeling of T cell subsets in healthy humans.

## Highlights

• Current best estimates of the proliferation and production of CD4^+^ and CD8^+^ T cell subsets.
• Explanation of why estimates vary between studies and which estimates have been superseded.
• Discussion of the implications of model choice.

## Graphical abstract

**Figure:**
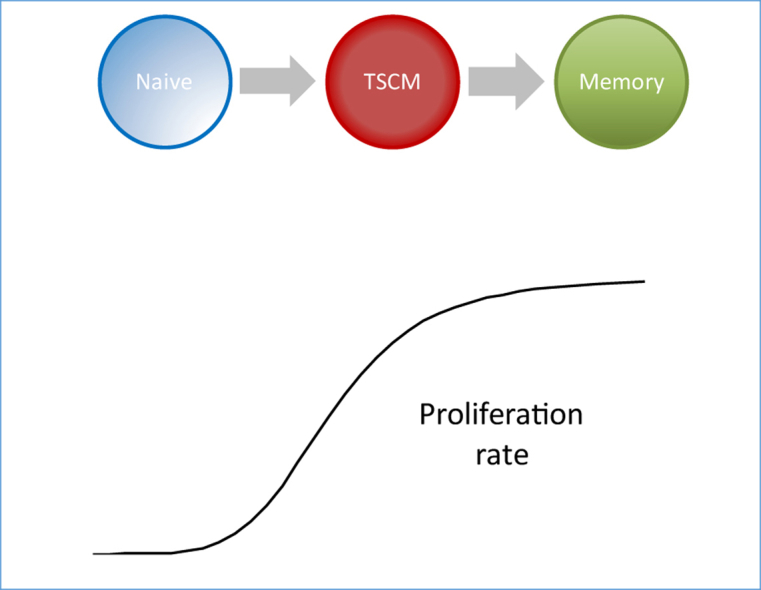

## Introduction

Recent advances in our understanding of human T cell kinetics have resulted from concurrent development of experimental and theoretical approaches. Experimentally, it has been difficult to find a methodology that reliably reports *in vivo* T cell kinetics. The earliest studies used the rate of loss of therapeutic X ray–induced chromosome changes in patients with ankylosing spondylitis [1] or cancer [2,3]. However, this method is only applicable to patients receiving radiotherapy and assumes that radiation damage does not impact cell lifespan. The next generation of quantitative studies in humans utilised the thymidine analogue bromodeoxyuridine (BrdU) [4] but this too was beset with difficulties, both ethical (BrdU is not without toxicities) and theoretical (BrdU incorporation in DNA may itself perturb cell lifespan [5,6]). The field was revolutionised in the 1990s by the advent of stable isotope labeling [7, 8, 9, 10]. Stable isotopes such as deuterium (^2^H) are thought to be nontoxic and to have no impact on cell lifespan at the tracer doses used [5,11]. The premise of this approach is that DNA synthesis is a surrogate for cell replication, that the cellular content of DNA is fixed and that nonreplicative DNA synthesis is negligible.

A cursory read of the stable isotope labeling literature can be confusing as the estimated kinetics (proliferation, generation, disappearance rates) of the various T cell subpopulations vary from one study to the next. This is in part due to evolving phenotypic definitions of T cell subsets, in part due to evolving modeling techniques and in part due to differences in nomenclature for the key parameters; even given these explanations some of the discrepancy remains unexplained. Here we review the quantification of T cell subpopulations with the aim of explaining the source of some of these differences between estimates, highlighting the current best estimates. We restrict ourselves to T cells in healthy humans.

## Stable isotope labeling: an overview

We start with a very brief overview of the stable isotope labeling method and then consider some of the important points in more detail later. A typical protocol for quantifying cell dynamics *in vivo* starts with the administration of the stable isotope label into a DNA precursor metabolite, typically either heavy water (^2^H_2_O) or deuterium-labeled glucose (6,6-^2^H_2_-glucose) [5,12]. Blood is sampled at regular time intervals, the cell populations of interest are sorted by fluorescence-activated cell sorting (FACS) and the fraction of labeled deoxyribose in their DNA is quantified by mass spectrometry. Label is gained when cells proliferate and lost when the labeled cell dies, differentiates to another phenotype or exits the blood compartment long-term. The time course of the fraction of labeled DNA thus contains information about cell proliferation and cell disappearance. To extract this information, mathematical models are constructed to describe the process and fitted to the experimental data. This permits the quantification of the rate of proliferation of any cell population that can be sampled and sorted in sufficient quantity in humans *in vivo*.

Two important considerations in labeling studies are (i) duration of labeling and (ii) data normalisation, both of which are closely related to the choice of label. For glucose, since the body pool is small and dynamic, ^2^H_2_-glucose enrichments peak quickly so labeling periods can be very short (bolus, 10 h, 24 h and 7 day durations have been used [13, 14, 15]). In contrast, because the body water pool is large, ^2^H_2_O labeling takes weeks. The lifespan of the cells of interest determines the optimal labeling period; ideally the target cell population should show considerable but not 100% replacement during the labeling period so that the signal is measurable but not saturated. Consequently, ^2^H_2_-glucose is good for rapidly dividing cells and D_2_O is better suited to slowly dividing populations.

In terms of normalisation, isotope labeling requires two normalisation steps: one to adjust for label availability in the individual (determined by dose, timing and dilution by unlabeled glucose/water), and one to scale between label in the plasma and the resulting label in DNA. For both water and glucose protocols, label availability in the plasma/body water is measured at multiple time-points and an empirical curve fitted to describe the enrichment, usually a square pulse with exponential tail for ^2^H_2_-glucose and a logistic growth/exponential decay curve for ^2^H_2_O. The second normalisation step is the scaling parameter (referred to as *c*, or *b*_*w*_ for ^2^H_2_O labeling and *b* or *b*_*g*_ for ^2^H_2_-glucose labeling); this is the ratio between label enrichment in newly synthesised DNA and that in plasma. *In vitro* ^2^H_2_-glucose labeling experiments show that enrichment levels in DNA plateau at about 60–75% of media enrichment [11,12]. This has been attributed to intracellular dilution by unlabeled preformed deoxynucleotide triphosphates and by other pentose precursors [11]. For ^2^H_2_O the scaling factor can be determined within the individual by sampling cells such as monocytes or granulocytes that can be expected to be fully replaced within the labeling period. By estimating their plateau enrichment, the scaling between DNA enrichment and plasma enrichment can be calculated. Deoxyribose contains seven nonexchangeable hydrogen atoms, any of which might potentially be replaced by deuterium in a D_2_O labeling study. Consequently, the enrichment seen in deoxyribose exceeds that seen in plasma. The scaling has a binomial dependence on label availability in the plasma (any of the sites can be labeled or not labeled with probability dependent on plasma label enrichment). It can be shown that when n = 7 then, for the plasma enrichments typically attained, the scaling factor would be expected to lie in the range 6.18-6.68 (not 7 as sometimes erroneously stated) [7]. Empirically the scaling factor is observed to lie in the range 3.2–5.2 [14,16]; why there is variation between individuals in what might be expected to be a basic biochemical parameter and why the observed range differs from the theoretically expected range is unclear and indicates that there may be errors in the calculation of this parameter. Label enrichment in DNA is directly proportional both to the scaling factor and to the proliferation rate (**Supplementary Information**) so an error in the scaling factor will cause the inverse error in the proliferation rate (if *b* is erroneously estimated to be double the true value, the proliferation rate estimated will be half the true value).

## T cell dynamics: what we have learnt

We focus on CD8^+^ T cells in the text and Table 1; analogous numbers for CD4^+^ T cells are provided in Table 2.

**Table 1:** Summary of estimates of CD8^+^ T cell generation and proliferation rates in humans.

Population	Phenotype	Generation rate (d^−1^)	Range (d^−1^)	Proliferation rate (d^−1^)	Range (d^−1^)	Source	Method	Notes
Naïve	CD45RA^+^	0.002 [100% self-renewal, 0% from thymus [19]]	0.0–0.01	0.002	0.0–0.01	Macallan et al. [13] Table 1^a^	1d ^2^H_2_-glu kh model	Based on best phenotypic definition at time. Will inadvertently have included T_EMRA_ and T_SCM_.
	CD45RO^―^CD27^+^	0.0003	0.0003–0.0005	0.0003	0.0003–0.0005	Vrisekoop et al. [16] Table 1^a^	63d ^2^H_2_O kh model	Based on best phenotypic definition at time. Will inadvertently have included T_SCM_.
	CD45RO^―^CD27^bright^CCR7^+^CD95^―^	**0.00045**	0.0003–0.0006	**0.00045**	0.0003–0.0006	Costa del Amo et al. [18] Table S2^a^	49d ^2^H_2_O precursor model	
T_SCM_	CD45RO^―^CD27^bright^CCR7^+^CD95^+^	**0.01** [64% self-renewal, 35% from naïve pool [18]]	0.006–0.07	**0.007**	0.002–0.01	Costa del Amo et al. [18] Table 1 and S2 (see notes)	49d ^2^H_2_O precursor model	Paper found evidence for ≥ two subpopulations. Here we report the average rates derived from Table 1 (ratio of subpopulations) and Table S2 (proliferation and disappearance of subpopulations)
Memory	CD45RA^―^			0.019	0.006–0.16	Macallan et al. [13] Table 1^¥^	1 d ^2^H_2_-glu kh model	Later corrected by Ahmed et al. see below
	CD45RO^+^			0.0028	0.0019–0.006	Vrisekoop et al. [16] Table 1^¥^	63d ^2^H_2_O kh model	Later corrected by Westera et al. and Ahmed et al. see below
	CD45RO^+^			0.006	0.004–0.009	Westera et al. [27] in line text (note typo on upper limit of range in original paper, corrected here)^¥^	63d ^2^H_2_O multi-exp model	Corrected analysis of Vrisekoop et al. data
	CD45RA^―^			**0.015**	0.006–0.11	Ahmed et al. [28] adjustment taken from Table S1 and applied to Macallan estimates^¥^	1d ^2^H_2_-glu kh model	Corrected analysis of Macallan et al. data.
	CD45RO^+^			**0.007**	0.004–0.009	Ahmed et al. [28] adjustment taken from Figure S7 (ratio of *b*_*w*_) and applied to Westera estimates ^¥^	63d ^2^H_2_O multi-exp model	Further corrected analysis of Vrisekoop et al. data.

Estimates (within each subpopulation) provided in chronological order. Estimates shown in bold font are current best estimates, estimates in regular font have been superseded (see final column, notes). All estimates, with the exception of the T_SCM_ subpopulation parameters, assume there is no input from a precursor population (Box 1). Work from Westera et al. indicates this is a valid assumption for naïve cells. However, for memory cells we cannot necessarily neglect input from the precursor population (in this case naïve cells). Proliferation rate estimates marked ^¥^ may therefore need to be revised when additional data are available.

All rates provided as proportion of target cell population and represent the median across study individuals.

Abbreviations: D_2_-glu: D_2_-glucose, kh model: kinetic heterogeneity model, multi-exp model: multi-exponential model.

Model equations provided in Supplementary Information.

The range is the minimum and maximum value of the point estimate observed across the subjects.

a Proliferation rate estimate for naïve cells calculated from production rate based on the finding that 100% of new naïve cells in humans originate from peripheral proliferation [19].

**Table 2:** Summary of estimates of CD4^+^ T cell generation and proliferation rates in humans.

Population	Phenotype	Generation rate (d^−1^)	Range (d^−1^)	Proliferation rate (d^−1^)	Range (d^−1^)	Source	Method	Notes
Naïve	CD45RA^+^	0.004	0.002–0.015	0.004	0.002–0.015	Macallan et al. [13] Table 1^a^	1d ^2^H_2_-glu kh model	Based on best phenotypic definition at time. Will inadvertently have included T_SCM_.
	CD45RO^−^CD27^+^	0.0005	0.0003–0.0009	0.0005	0.0003–0.0009	Vrisekoop et al. [16] Table 1.^a^	63d ^2^H_2_O kh model	Based on best phenotypic definition at time. Will inadvertently have included T_SCM_.
	CD45RO^―^CD27^bright^CCR7^+^CD95^―^	**0.0007**	0.0004–0.001	**0.0007**	0.0004–0.001	Costa del Amo et al. unpub.	49d ^2^H_2_O precursor model	
T_SCM_	CD45RO^―^CD27^bright^CCR7^+^CD95^+^	NA	NA	NA	NA			Parameters not estimated for CD4^+^ T_SCM_ due to lack of additional data (YFV)
Memory	CD45RA^―^			0.02	0.01–0.08	Macallan et al. [13] Table 1^¥^	1d ^2^H_2_-glu kh model	Later corrected by Ahmed et al. see below
	CD45RO^+^			0.0045	0.002–0.007	Vrisekoop et al. [16] Table 1^¥^	63d ^2^H_2_O kh model	Later corrected by Westera et al. and Ahmed et al. see below
	CD45RO^+^			0.0061	0.002–0.01	Westera et al. in line text [27]^¥^	63d ^2^H_2_O multi-exp model	Corrected analysis of Vrisekoop et al. data
	CD45RA^―^			**0.018**	0.009–0.05	Ahmed et al. [28] adjustment taken from Table S1 and applied to Macallan estimates^¥^	1d ^2^H_2_-glu kh model	Corrected analysis of Macallan et al. data.
	CD45RO^+^			**0.0064**	0.002–0.01	Ahmed et al. [28] adjustment taken from Figure S7 (ratio of *b*_*w*_) and applied to Westera estimates ^¥^	63d ^2^H_2_O Multi-exp model	Further corrected analysis of Vrisekoop et al. data.
Central memory	CD45RO^+^CCR7^+^			**0.010**	0.007–0.04	Macallan et al. [43] Table 2^¥^	1d ^2^H_2_-glu kh model	
Effector memory	CD45RO^+^CCR7^-^			**0.042**	0.02–0.08	Macallan et al. [43] Table 2^¥^	1d ^2^H_2_-glu kh model	

Estimates (within each subpopulation) provided in chronological order. Estimates shown in bold font are current best estimates, estimates in regular font have been superseded (see final column, notes). All estimates assume there is no input from a precursor population (Box 1). Work from Westera et al. indicates this is a valid assumption for naïve cells. However, for memory cells we cannot necessarily neglect input from the precursor population (in this case naïve cells). Proliferation rate estimates marked ^¥^ may therefore need to be revised when additional data are available.

The range is the minimum and maximum value of the point estimate observed across the subjects.

All rates provided as proportion of target cell population and represent the median across study individuals.

Abbreviations: D2-glu: D2-glucose, kh model: kinetic heterogeneity model, multi-exp model: multi-exponential model.

Model equations provided in Supplementary Information.

a Proliferation rate estimate for naïve cells calculated from production rate based on the finding that 100% of new naïve cells in humans originate from peripheral proliferation [19].

**Naïve CD8**^+^ **T cells.** Thymic output and peripheral proliferation both contribute to maintenance of the naïve T cell pool. Since the isotope will label any proliferating cell, no distinction can be made between cells that divided in the periphery and cells that acquired label in the thymus and then entered the periphery [17]. Most models to describe naïve cell dynamics ignore thymic output and attribute all label accrual to naïve cell proliferation; this will tend to lead to an overestimate of proliferation rates and an underestimate of disappearance rates (Box 1). This caveat aside, the first study to investigate naïve T cells using isotope labeling found a median naïve CD8^+^ T cell proliferation rate of p = 0.002 d^−1^ (corresponding to a doubling time of ln(2)/0.002 = 295 days) [13], Table 1, Table 2. A later study found a considerably slower proliferation rate (p = 0.0003 d^−1^, doubling time = 2300 days) [16]. Part of this difference can be explained by the problem of evolving phenotypic definitions. Both studies inadvertently included what we would now call non-naïve cells in their sorted “naïve” populations (defined as CD45RA^+^ and CD45RO^―^CD27^+^, respectively). This would have included terminally differentiated effectors (T_EMRA_) in the first study and stem cell memory T cells (T_SCM_) in the second (neither of which had been described at the time). A study using the most recent definition (CD45RO^―^CD27^bright^CCR7^+^CD95^―^) finds that naïve CD8^+^ T cells have a proliferation rate of 0.0005 d^−1^ (doubling time = 1400 days) [18]. By including an analysis of T cell receptor excision circles (TRECs), a further study split naïve CD4^+^ T cell production into cells arising from the thymus and cells arising from peripheral proliferation. It was shown that in adult humans, in stark contrast to mice, the overwhelming majority of new naïve CD4^+^ cells (approx. 100%) arose from peripheral division [19]. This fascinating result illustrates the importance of studying humans wherever possible. Unfortunately, although T_SCM_ cells had been described by this point, the analysis used an older definition of naïve T cells (CD45RO^―^CD27^+^) which would have included T_SCM_ cells. True naïve cells have lower proliferation and much higher TREC content than T_SCM_ cells [18,20,21]; thus a large proportion of peripheral proliferation, which was attributed to naïve cells in this study, may in fact be due to T_SCM_ cells. Moreover, T_SCM_ cells are a considerably more frequent population in humans than mice and so the failure to exclude T_SCM_ cells from the naïve cell gate may partly explain why more replacement by proliferation was seen in humans than mice [20,22]. It would be interesting to redo this important analysis delineating the contribution of true naïve cells and T_SCM_ cells. It seems likely that the inclusion of T_SCM_ cells could explain some but not all of the reported difference between mice and humans in the contribution of thymic output.

Box 1 **Nomenclature: generation, proliferation, disappearance, turnover and half-lives.Image 6**

For a target population in equilibrium (i.e. at steady state), the generation of new cells (at rate *g*) either by influx from a precursor (progenitor) population at rate *i* and/or proliferation (i.e. self-renewal) at rate *p*, is balanced by cell disappearance (death, differentiation, long-term exit from blood, at rate *d*) such that *g*=*i*+*p*=*d*. With a few exceptions [14,18,24,45], the majority of labeling studies ignore the influx from precursor populations (i.e. *i* is assumed to be zero) and all label accrual is attributed to self-renewal. The impact of this assumption on the rates estimated will depend on both the kinetics and size of the precursor population compared with the target population; the impact of including an upstream compartment has been demonstrated explicitly for neutrophils [14], CD4^+^ memory T cells [24] and CD4^+^ and CD8^+^ T_SCM_ cells (unpublished work).

The expansion of models to include a nonzero influx term means that two widely used terms in cell kinetics, turnover and half-life, which were previously precisely defined, are now ambiguous. A number of studies report the “turnover rate” of a population which is either the proliferation rate or the disappearance rate. When the influx from the precursor population is assumed to be zero then, for a population at steady state, the proliferation rate equals the disappearance rate and the turnover rate is unambiguously defined. However, when the influx is nonzero, the definition of “turnover” is unclear and we avoid its use in this review. Similarly for the population half-life. With zero influx the half-life (time for the population to halve in size if there was no proliferation) = ln(2)/p = ln(2)/d. However, if nonzero influx is considered, then another parameter can be defined, the “clonal half-life”, ln(2)/(d-p). The clonal half-life is the time for a T cell clone to halve in size whilst the overall population remains at steady state (i.e. without halting proliferation) and likely relates to the longevity of a T cell clone (for zero influx the clonal half-life is infinite). Models describing an upstream/precursor population and a downstream/target cell population were used by Costa del Amo et al. to describe the relationship between naïve and T_SCM_ cells [18]; the equations are given in **Supplementary Information**.

This input from a precursor population considered earlier is distinct from the “source” term included in early models of labeling dynamics [46,47]. In these early models the target cell population was assumed to be homogeneous and the source was invoked to explain the observation that the measured disappearance rate typically exceeded the measured proliferation rate despite the fact that the target cell population was of approximately constant size over time. However, the size and nature of the source in these models (typically large and unlabeled) meant it was usually not possible to find a physiological correlate of the source. Instead it was proposed that the discrepancy between the measured proliferation and disappearance rates could be explained by kinetic heterogeneity in the population [17]. That is, if the target population is heterogeneous, then although the measured proliferation rate will be the average proliferation rate of the population the loss of labeled cells will be biased towards the rapidly turning over subpopulation and will therefore overestimate the average disappearance rate of the whole population.

**Table:** Definitions

Proliferation	p
Disappearance	d
Doubling Time	ln(2)/p
Half-life	ln(2)/d
Clonal half-life	ln(2)/(d-p)
Lifespan	1/d

The above-mentioned definitions are correct under the assumption that times to proliferate and disappear are exponentially distributed.

Alt-text: Box 1

**Stem cell memory T cells (T**_SCM_**).** T_SCM_ cells are a recently described subpopulation of T cells with stem cell–like properties of self-renewal, clonal longevity and multipotency. It is postulated that they are responsible for maintaining long-lived immune memory [20]. Until 2018, self-renewal and longevity of human T_SCM_ cells had only been demonstrated *in vitro*. Using a combination of mathematical modeling, stable isotope labeling, telomere length analysis and data from vaccinees, it was possible to quantify the self-renewal, proliferation and clonal longevity of T_SCM_ cells in humans *in vivo*. Unexpectedly, it was found that the average lifespan of a T_SCM_ clone is short (clonal half-life < 1 year, proliferation rate = 0.007 d^−1^), far too short to maintain immune memory that can last for decades. However, it was also shown that what we currently define as the T_SCM_ population (CD45RO^―^CD27^bright^CCR7^+^CD95^+^ [23]) comprises at least two kinetically distinct subpopulations. One is rapidly replaced (clonal half-life ln(2)/(d-p) = 5 months) which explains the short average lifespan of the bulk T_SCM_ population, and the other having a clonal half-life of approximately 9 years, consistent with the longevity of immune memory. This long-lived subpopulation had a high degree of self-renewal, with a cell residing without dying or differentiating for 15% of our lifetime. It was postulated that this subpopulation represents the “true” stem cell–like population (the other subpopulation may represent cells transiting to effector status). Interestingly, in apparently healthy asymptomatic individuals, there was ongoing differentiation of naïve cells; the contribution of naïve cells to T_SCM_ replacement was typically about 50% and never less than 10% (i.e. 10–50% of new T_SCM_ cells are produced by differentiating naïve cells and 50–90% by division of existing T_SCM_ cells). Considerable recruitment of naïve cells to the memory pool in the apparent absence of novel antigen has previously been reported for mice [24,25]. To the best of our knowledge this is the first time this surprising observation has been made in humans, and it is important to confirm it using an independent approach.

**Memory T cells.** Only one stable isotope labeling study of CD8^+^ T cell memory subpopulations (central memory and effector memory) has been carried out [26]. In this study, sampling was restricted to long after the end of labeling, and thus only quantifies the net loss of labeled cells and does not permit the separation of proliferation and disappearance. It is necessary to sample both the uptake and the loss of label to obtain a representative estimate of proliferation [17]. To date this has only been performed for bulk (CD45RO^+^ or CD45RA^−^) CD8^+^ memory T cells (though some CD4^+^ memory subsets have been studied, Table 2). The first such study, which used a 24 h ^2^H_2_-glucose labeling protocol, found that memory CD8^+^ T cells proliferated rapidly (p = 0.019 d^−1^, doubling time = 36 days). A subsequent study, utilising a 63 d D_2_O protocol, found considerably slower proliferation (p = 0.0028 d^−1^, doubling time = 248 days).

Investigation of this discrepancy by a collaboration between the two groups involved [27,28] has highlighted some critical factors in analysing isotope labeling data. Firstly, in long labeling studies, saturation of subpopulations must be accounted for. Adjusting for saturation in the 63-day ^2^H_2_O study led to revised estimates of p = 0.006 d^−1^ (doubling time = 116 days). Whether saturation has occurred can be investigated by comparing the fits of models with increasing numbers of subpopulations and checking for a change in the estimated average proliferation rate [27,29]. Secondly, normalisation is critical, particularly for overnight ^2^H_2_-glucose labeling. We postulated that errors may arise from reduced plasma glucose sampling overnight when unlabeled glucose influx (food) is reduced; periods of higher glucose enrichment may thus be missed. A prediction of this postulate (that monocytes would have plateau label of >100%) was borne out. Correcting for these inaccuracies in normalisation decreased proliferation rate estimates made using D_2_-glucose (to p=0.015 d^-1^) and increased estimates made using D_2_O (to p=0.007 d^-1^). A more sophisticated approach in which plasma glucose was predicted rather than measured came to very similar conclusions [30]. The estimates from the two studies are now closer together, but there does still appear to be some genuine, unresolved difference. Current best estimates of the proliferation rate of memory CD8^+^ T cells are therefore in the range 0.007-0.015 d^−1^ (corresponding to a doubling time of between 46 and 99 days).

**Figure 1:**
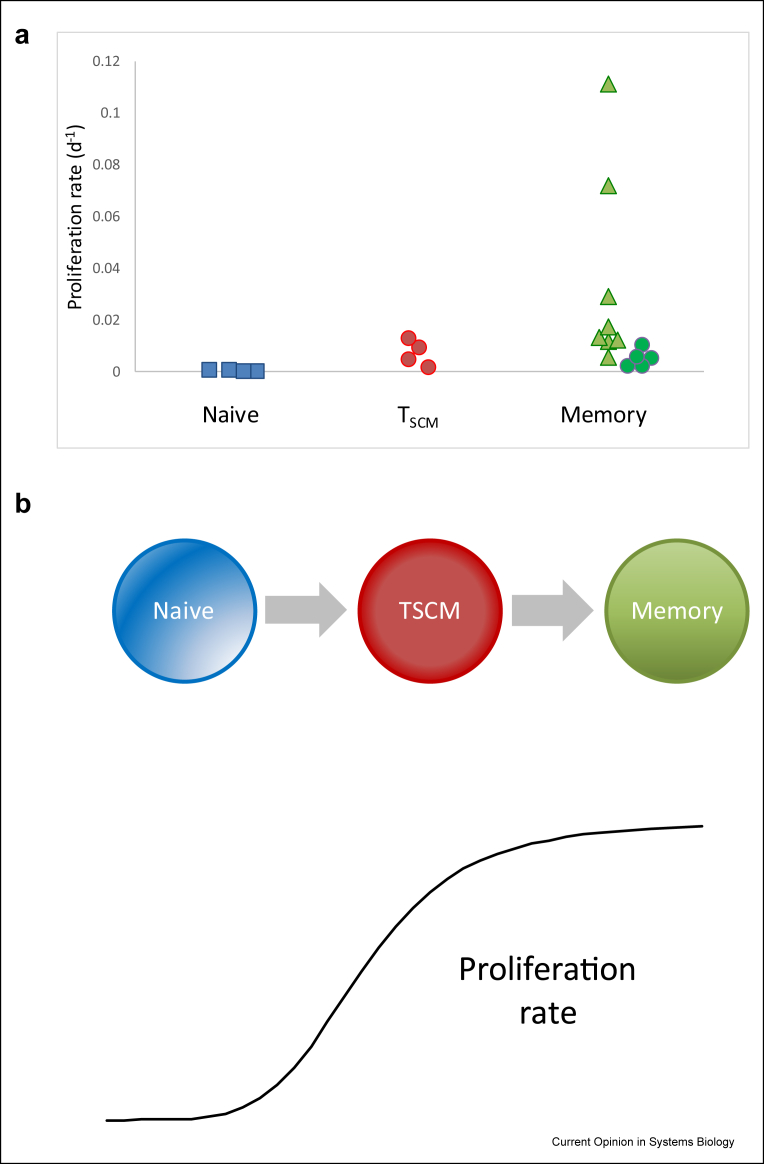
**Estimates of CD8**^+^**T cell proliferation rates as a function of cell differentiation state**.**a**. Current best estimates of the proliferation rates for different CD8^+^ T cell subsets in healthy adult humans (Table 1). Naïve T cells. Estimates from Costa del Amo et al. [18]. T_SCM_ cells. Estimates from Costa del Amo et al. [18]. Memory T cells. Estimates from Macallan et al. adjusted by Ahmed et al. [13,28]. Memory T cells. Estimate from Vrisekoop et al. adjusted by Westera et al., further adjusted by Ahmed et al. [16,28,44].**b** Cartoon of proliferation rate as a function of differentiation state. All the evidence indicates that naïve T cells proliferate more slowly than T_SCM_ cells and memory T cells, i.e. estimated T cell proliferation increases with differentiation despite the decrease in proliferative potential that is thought to be associated with differentiation [23,31,32].

It is interesting that these studies show that more highly differentiated T cells have more rapid proliferation (Fig. 1; see also CD4^+^ subpopulations Table 2) despite the decrease in proliferative potential that is thought to be associated with differentiation [23,31] and the prevalent view that cell senescence is linked with poor proliferation [32, 33, 34].

## Modeling considerations

Mathematical models of T cell kinetics necessarily involve assumptions, firstly because we do not completely understand the biology (e.g. kinetic substructure of T cell populations [17,24], the lineage relationship between T cell subpopulations [35], the rules determining cell fate [36, 37, 38∗∗]) but secondly, more importantly, even if the biology was completely known, it would still be essential to simplify models for parameter inference (if models are overly complex compared with the data, the parameters cannot be estimated). And so mathematical models of T cell dynamics for parameter inference will always contain assumptions. This means that estimates from isotope labeling are necessarily susceptible to model assumptions. This problem was starkly illustrated by a recent debate over the lifespan of neutrophils. In 2012, a paper by Pillay et al. utilising isotope labeling found that, far from being a very short-lived population as previously thought, blood neutrophils had lifespans of about 5 days, at least 10 times longer than previous estimates [39, 40]. If correct, the work by Pillay et al. would have overturned two decades of research. However, a subsequent response proposed that the unexpectedly long lifespans from the labeling study could result from data misinterpretation [41]. This was later confirmed by Lahoz-Beneytez et al. who combined a reanalysis of the data from Pillay et al. as well as generation of new labeling data to show that the 2012 labeling study had included an implicit unphysiological assumption. Lahoz-Beneytez et al. showed that by neglecting the fact that neutrophils are produced by proliferation outside of the observed compartment (i.e. are produced by proliferating precursors in the bone marrow rather than by proliferation of neutrophils in the blood), Pillay et al. had essentially assumed the ratio of blood neutrophils to the bone marrow neutrophil precursor mitotic pool was infinite, leading to a severe underestimate of the neutrophil death rate [14]. Using a more physiological model, Lahoz-Beneytez et al. found the best estimate of neutrophil half-life lay in the range 13–19 h, consistent with the traditional dogma that blood neutrophils are short-lived. More generally, neglecting the upstream compartment will affect kinetic estimates of all cell populations (Box 1). Proliferation rates of memory T cells (currently estimated without allowing for input from the naïve T cell compartment) are also susceptible to such error and may need to be revised once more data are available [18,24].

## Future directions

There are a number of areas that are ripe for development. The first area where more research is essential is robustness of parameter estimates to model assumptions. Personally, we routinely examine the impact of assumptions that we are conscious of making (e.g. neglecting recirculation) on our parameter estimates, but this approach is very limited and *ad hoc*. The problem is compounded because only a few groups work on isotope labeling data and we collaborate closely so there is a lack of independent criticism that comes from having many researchers working independently on the same problem. One way forward may be to use the ideas of parameter estimation from ensemble models that are being developed in the field of climate change modeling; e.g., Ref. [42]. Another area where progress is lacking is the normalisation factor for D_2_O labeling, *c* (*b*_*w*_): why does this vary between individuals and why does it frequently lie outside the range that would be theoretically expected? An error in normalisation will directly affect proliferation rate estimates and, to a lesser extent disappearance rate estimates. The field has been aware of this problem for more than 10 years yet has failed to progress. And then there are a number of easier problems: “what is the proliferation rate of the various CD8^+^ T cell subpopulations that have yet to be studied e.g. effector memory, central memory, T_EMRA_?“, “is the statement that all naïve cell replacement comes from peripheral proliferation in humans true if we exclude T_SCM_ cells from the naïve cell gate?“, “what happens to our current estimates of memory T cell proliferation if we include input from differentiating naïve T cells?” In short, there has been a decade of considerable progress, but there are still many fundamental questions to be answered and we would urge the community to consider addressing these topics.

## Conflict of interest statement

Nothing declared.
