# Supplementary material for: Current estimates of T cell kinetics in humans
Source: Curr Opin Syst Biol. 2019 Dec;18:77–86. doi: 10.1016/j.coisb.2019.10.002 (PMC6941842; doi:10.1016/j.coisb.2019.10.002)
Supplement: Multimedia component 1 [file mmc1.docx]

# Supplementary Information: Summary of Mathematical Models

In **Table 1** we refer to the different mathematical models used to estimate T cell kinetics. Here we summarise the models.

In all equations below *b_w/g_* is the normalisation constant that scales from label enrichment in plasma to label enrichment in DNA for ^2^H_2_O and D_2_-glucose respectively (*b_w_*=3.2-5.2, *b_g_*=0.65-0.73) and *U(t)* is a function describing label enrichment in plasma.

**Kinetic heterogeneity model**

Underling cell population model for cell population *T* proliferating at rate *p* and disappearing (dying, differentiating, exiting blood long-term) at rate *d*:

At steady state *p*=*d.*

Fraction of labeled deoxyribose is *F:*

where *d** is the disappearance rate of labeled cells (in general for a kinetically heterogeneous subpopulation *d*≠*d* [17]*). Of the estimates reported in **Table 1** & **2**, this model was used by [13] and [16].

**Multi-exponential model**

Underling model for cell population consisting of two subpopulations *T_1_* and *T_2_* of size α and (1-α) respectively. Cells in *T_1_* proliferate at rate *p_1_* and disappear (die, differentiate, exit blood long-term) at rate *d_1_;* cells in *T_2_* proliferate at rate *p_2_* and disappear at rate *d_2_*:

At steady state *p_1_*=*d_1_, p_2_=d_2_*

Fraction of labeled deoxyribose in the whole population coming from subpopulation *T_1_* (*T_2_*) is *F_1_ (F_2_),* assuming steady state*:*

The sum *F_1_*+*F_2_* is fitted to the observed fraction of label in the population. Note, *α*, *p_1_* and *p_2_* are not identifiable which may cause problems for fitting routines; however the average proliferation *αp_1_+(1-α)p_2_* is identifiable. Of the estimates reported in **Table 1** & **2** this model was used by [27].

**Precursor model (implicit heterogeneity version)**

Underlying model for a cell population *T_N_* that proliferates at rate *p_N_*, disappears at rate *d_N_* and differentiates at rate *Δ*. Differentiation is accompanied by *k* rounds of clonal expansion. *T_N_* differentiate into *T_S_*. *T_S_* proliferate at rate *p_S_* and disappear at rate *d_S_*:

The fraction of labelled deoxyribose in the two populations is:

Where *R=T_N_/T_S_* (i.e. the relative size of the two populations), *d_N_^*^* is the disappearance rate of labelled *T_N_* cells and *d_S_^*^* is the disappearance rate of labelled *T_S_* cells.

**Precursor model (explicit heterogeneity version)**

Underlying model for a homogenous cell population *T_N_* that proliferates at rate *p_N_*, disappears at rate *d_N_* and differentiates at rate *Δ*. Differentiation is accompanied by *k* rounds of clonal expansion. *T_N_* differentiate into *T_S_* which is heterogeneous and consists of two subpopulations *T_S1_* and *T_S2_*. A fraction (1-*f)* differentiate into *T_S1_* and the remainder *(f)* differentiate into *T_S2_*. *T_S1_* proliferate at rate *p_S1_* and disappear at rate *d_S1,_* similarly for *T_S2_*:

Assuming steady state of the three populations *T_N_*, *T_S1_* and *T_S2_* independently, the fraction of labelled deoxyribose in the three populations is:

Where *R_1_=T_N_/T_S1_* (i.e. the relative size of the precursor population to the first subpopulation) and *R_2_=T_N_/T_S2_* (i.e. the relative size of the precursor population to the second subpopulation). Of the estimates reported in **Table 1** & **2** this model was used by [18].

**Relationship between the models**

The kinetic heterogeneity model and multi-exponential model both consider a single, closed population of cells with no input from a precursor population; cells can proliferate or disappear. Both models allow this population to be kinetically heterogeneous i.e. to consist of subpopulations with different kinetics. In the kinetic heterogeneity model this heterogeneity is dealt with implicitly by allowing *d*≠*d**; in the multi-exponential model the heterogeneity is dealt with explicitly by describing the two subpopulations. The multi-exponential model suffers from parameter identifiability problems due to the strong correlation between the size of a subpopulation and its proliferation rate (i.e. a range of values of *α*, *p_1_* and *p_2_* give the same labelling curves and so are not distinguishable) this means that fitting routines will struggle to converge (there is no unique solution) and the individual parameters cannot be identified; however the average proliferation *αp_1_+(1-α)p_2_* is identifiable. In the absence of saturation the average proliferation from the multi-exponential model and the proliferation from the kinetic heterogeneity model are equivalent.

The precursor models (implicit heterogeneity version and explicit heterogeneity version) are the extensions of the kinetic heterogeneity model and the multi-exponential model respectively to describe an upstream precursor population differentiating into the target population of interest. The versions presented here were developed to describe naïve cells differentiating into T_SCM_ cells. We showed that there was no reason to reject homogeneity for the naïve cell population but that there was strong evidence to reject it for the T_SCM_ population [18] and so the models here assume that the precursor population is homogeneous but allow for heterogeneity (implicitly or explicitly) in the downstream T_SCM_ population. It has been shown (Mackerodt unpub) that both models can be reparameterised to improve parameter identifiability but there are still problems with fitting the explicit version just to labelling data. In our experience it is necessary to include additional data (we used telomere length and cell numbers) to adequately constrain the parameters.
